# Effects of different cutting methods and additives on the fermentation quality and microbial community of *Saccharum arundinaceum* silage

**DOI:** 10.3389/fmicb.2022.999881

**Published:** 2022-09-23

**Authors:** Yulong Zheng, Mengxin Li, Jinyi Xu, Hong Sun, Qiming Cheng, Yixiao Xie, Chunmei Wang, Chao Chen, Ping Li

**Affiliations:** ^1^Collage of Animal Science, Guizhou University, Guiyang, Guizhou, China; ^2^Key Laboratory of Animal Genetics, Breeding and Reproduction in the Plateau Mountainous Region, Ministry of Education, Guizhou University, Guiyang, Guizhou, China

**Keywords:** silage, *Saccharum arundinaceum*, *Lactobacillus plantarum*, cellulase, cutting methods, bacterial community

## Abstract

To develop a new high-yielding and polysaccharide-containing forage resource for livestock, the effects of different cutting methods and additives on *Saccharum arundinaceum* silage were evaluated. The wilted *S. arundinaceum* were chopped and knead-wired. The silages from each cutting method were treated with *Lactobacillus plantarum* (LP), cellulase (CE) and the combination of LP and CE (LP + CE) for 3, 7, 15, 30, and 60 days. Compared with the CK treatment, CE treatment exhibited better effects in the degradation of neutral detergent fiber (NDF), LP exhibited a better performance in preserving the content of dry matter (DM), and adding LP + CE significantly enhanced (*P* < 0.05) the contents of lactic acid (LA), crude protein (CP) and DM and significantly reduced (*P* < 0.05) the pH and NDF content during ensiling. In addition, both additives exerted a remarkable effect on the silage bacterial community (*P* < 0.05), with a dramatic increase in the *Lactobacillus* abundance and a decrease in the abundance of *Enterobacter*. Lactic acid bacteria (LAB) became the most dominant bacteria that affected the fermentation quality of LP and LP + CE silages. Meanwhile, chopped silages showed better fermentation quality and nutrient preservation and a higher abundance of LAB. Our research indicated that the chopped *S. arundinaceum* ensiling with LP + CE could exert a positive effect on LA fermentation and preservation of nutrient substances by shifting the bacterial community. In conclusion, *S. arundinaceum* can serve as a new silage resource for feed utilization by the ensiling method of LP + CE-chopped.

## Introduction

*Saccharum arundinaceum* is a tall-growing, caespitose perennial herbaceous grass native to southern China, parts of South and Southeast Asia and other countries (Wang W. G. et al., 2019). *S. arundinaceum* is a C4 plant and produces large amounts of biomass, for which the dry matter yield is approximately 40–60 t⋅ha^–1^⋅year^–1^; lignocellulosic biomass includes a high content of cellulose and polysaccharides (Heaton et al., 2008; Hattori and Morita, 2010). Therefore, *S. arundinaceum* has received increasing attention as a potential biofuel feedstock (Kirupa et al., 2018; Muthuvelu et al., 2018; Zhang et al., 2020). The high biomass yield and high polysaccharide content indicate that *S. arundinaceum* can be a potential feed source (Muthuvelu et al., 2018). In addition, the feeding value of *S. arundinaceum* can be increased through certain processing methods (such as ensiling). However, to our knowledge, research on *S. arundinaceum* has been mainly focused on its ecological restoration and biomass energy (Muthuvelu et al., 2018; Zhao et al., 2020). Few studies have paid attention to its feeding value and improving its quality through ensiling.

Ensiling is a process of rapid anaerobic fermentation of plant material by either epiphytic or exogenously applied lactic acid bacteria (Cai et al., 1998; Ren et al., 2020). Silage is one of the most important ruminant feedstocks in China, particularly southern China, where local climactic conditions make traditional hay production impractical (Dunière et al., 2013; Du et al., 2021). However, low amounts of epiphytic LAB and water-soluble carbohydrate (WSC) limit the applicability of some forages for ensiling (Xu et al., 2020; Du et al., 2021). Therefore, biological additives, including *Lactobacillus plantarum* and cellulase, can be crucial for improving the fermentation and nutrients of silage (Xu et al., 2018; Cai et al., 2020; Guo et al., 2020). Previous studies showed that adding *L. plantarum* could decrease the DM loss, pH, ammonia-N (NH_3_-N) content and butyric acid (BA) concentration, increase the LA concentration and change the bacterial community composition of silage (Ren et al., 2020; Xu et al., 2020; Zhang et al., 2021). Moreover, ensiling with cellulase could degrade part of the cellulose, thus providing a large amount of WSCs for LAB utilization of *S. arundinaceum* silage (Feng et al., 2020; Xu et al., 2020). Therefore, it is inferred that *S. arundinaceum* could potentially be used as a feed source for animals if *L. plantarum* and cellulase were applied during ensiling.

Cutting is used to obtain the appropriate particle size of the raw material, which is a necessary step in silage processing technology and an important basis to ensure the quality of silage (Savoie et al., 1992; Thomson et al., 2017). Different raw materials may have different optimal particle sizes by different cutting methods, such as chopping and knead wiring, due to the physical characteristics of the crop stems (Savoie et al., 1992; Sun et al., 2020). In general, raw materials of conventional silage are chopped into 1∼3 cm lengths by a straw crusher (Dong et al., 2020; Ren et al., 2020; Wang et al., 2021). Knead wire uses a kneading machine to flatten, shred, beat, tear and crush the forage grass, which is then kneaded and cut into soft silks. However, to the best of our knowledge, the effect of chopping and knead-wiring on silage fermentation with a high content of lignocellulosic biomass is unclear. Moreover, we hypothesized that the two cutting methods would have different effects on *S. arundinaceum* silage fermentation and the microbial community.

Therefore, chopped and knead-wired *S. arundinaceum* were ensiled with different additives in our study. The silage quality and microbial community were explored to evaluate the optimal cutting methods and additives. This study lays a research foundation for the development of new forage resources for animal production.

## Materials and methods

### Silage material production

Both the tender stems and leaves of *S. arundinaceum* were harvested from Sichuan Province, China. The raw materials were allowed to wilt until they reached approximately 70% moisture and then cut using two different methods: (1) a straw crusher was used to chop material into lengths of 1∼2 cm; or (2) a kneading machine was used to knead-wire the material. *Lactobacillus plantarum* was previously isolated by our research team. Cellulase was obtained from Yakult (Yakult Pharmaceutical Industry Co., Tokyo, Japan). All additives were added to fresh *S. arundinaceum* material after cutting.

Our 2 × 4 × 5 experiment was performed with a completely randomized design: 2 cutting methods (chopping, knead-wiring) × 4 additive treatments [1.0 × 10^6^ colony forming units (cfu)/g FM (Fresh matter, FM) LP, 50 U/g FM CE, 1 × 10^6^ cfu/g FM LP + 50 U/g FM CE (LP + CE), and the control (CK)] × 5 fermentation periods (3, 7, 15, 30, and 60 day). Three polyethylene bags (300 mm × 230 mm) were loaded with 500 g of silage and vacuum sealed per treatment, for a total of 120 experimental bags. All bags were stored at room temperature (approximately 25°C). Three bags for each treatment were opened to examine fermentation quality and microbial population at days 3, 7, 15, 30, and 60 during ensiling. The chemical components were evaluated after 60 day of fermentation. The bacterial community was analyzed after 60 day of ensiling.

### Chemical analyses

To measure fermentation indices, 10 g of each silage sample was blended with 90 mL of sterilized water and then filtered through qualitative filter paper. The silage pH and contents of NH_3_-N and organic acids were then determined using the resulting filtrate. The pH was immediately measured with a PHS-3C pH meter equipped with a glass electrode (INESA Scientific Instrument Co., Shanghai, China). Then, the filtrate was frozen in −20°C storage prior to determining the concentration of NH_3_-N and organic acids. The NH_3_-N concentration was quantified using the phenol-hypochlorite method proposed by Broderick and Kang (1980). The concentrations of various organic acids, including acetic acid (AA), BA, propionic acid (PA), and LA, were determined through high-performance liquid chromatography (HPLC) (LC-20A, Shimadzu, Tokyo, Japan) (Zhang et al., 2016).

To quantify the silage DM content, the samples were dried at 65°C in an air circulation oven for 48 h. Then, the samples were pulverized and passed through a 1 mm screen. The Van Soest et al. (1991) method was employed to determine both acid detergent fiber (ADF) and NDF. The Association of Official Agricultural Chemists [AOAC] (1995) method was employed to determine the content of CP. Murphy (1958) anthrone method was used to quantify the WSC content.

### Investigation of microbial populations

The plate count method (Li P. et al., 2019) was employed to quantify the size of silage microbial populations. Briefly, each 10 g sample was mixed 1:9 (w/v) with sterilized water, followed by serial dilution from 10^–1^ to 10^–6^ using sterilized water. Yeast population densities were enumerated by plating on Rose Bengal agar (YM01435, Shyuanmu Biomart Biotech. Co., Shanghai, China) containing 1.5 mg/L tetracycline, and LAB population densities were enumerated by De Man, Rogosa and Sharpe (MRS) agar (GCM188, Beijing Land Bridge Technology Co., Beijing, China). Yeasts and LAB were incubated for 72 h at 30°C and for 48 h at 37°C, respectively. Each plate count was transformed into log_10_ cfu/g FM.

### Analysis of bacterial community structure

Total bacterial DNA was extracted from 60-day silage samples according to the sodium dodecyl sulfate (SDS)/cetyltrimethylammonium bromide (CTAB) method. We used 1% agarose gels to assess DNA concentration and purity and extracted DNA diluted with sterilized water to a standardized concentration of 1 ng/μL. We amplified the bacterial 16S rRNA genes with the broad-range primers 27F (5-GAGAGTTTGATC CT GGCTCAG-3) and 1541R (5-AAGGAGGTG ATCC AGCCGCA-3) (Yan et al., 2019). TransStart^®^ FastPfu DNA Polymerase (TransGen Biotech, Beijing, China) was used to carry out polymerase chain reactions (PCRs). The same volume of 1X loading buffer was mixed with PCR products, and electrophoresis was performed on a 2% agarose gel for detection. The QIAquick@ Gel Extraction Kit (Qiagen, Hilden, Germany) was used for PCR product purification. The SMRTbell™ Template Prep Kit (Pacific Biosciences, Menlo Park, CA, USA) was used to generate the SMRTbell library following the manufacturer’s standard instructions. The Qubit@ 2.0 Fluorometer (Thermo Fisher Scientific, Waltham, MA, USA) and a FEMTO Pulse system were used to ensure library quality.

Raw sequences were processed, and a sequencing library was created by Novogene Bio Technology Co. (Beijing, China) using the PacBio Sequel sequencer (Pacific Biosciences, Menlo Park, CA, USA). Taxonomic information was annotated in the Silva SSUrRNA Database.^1^ After determining the phylogenetic relationships, the Magic platform was employed to calculate alpha diversity (Chao 1, Shannon, and Ace) and coverage values and perform bacterial community functional prediction (Li et al., 2021).

### Data processing and statistical analyses

SPSS ver. 23.0 for Windows (SPSS Inc. Chicago, IL, USA) was employed to carry out all statistical analyses. One- and two-way analyses of variance (ANOVA) were carried out using the fixed effects of additives, cutting methods, and ensiling days. To assess the separation of means under different fermentation additives, cutting methods, and fermentation times, Duncan’s multiple range test was performed. *P* < 0.05 was set as the threshold of statistical significance. Heatmaps based on Spearman correlation coefficients between the bacterial population and fermentation quality and function prediction were calculated with SPSS software. All data are reported as the mean with statistical significance indicated with letters. All figures were generated using OriginPro 2017.^2^

## Results and discussion

### Characteristics of fresh *Saccharum arundinaceum* before ensiling

The characteristics of the fresh material before ensiling are presented in Table 1. The content of DM in fresh *S. arundinaceum* was 284.2 g/kg. The CP content of *S. arundinaceum* was 104.13 g/kg on a DM basis, which was similar to that of oats (Zhao et al., 2018). However, the relatively high contents of NDF (672.51 g/kg DM) and ADF (302.32 g/kg DM) and the low content of WSCs (g/kg DM) are not conducive to the fermentation of LAB and animal utilization (Xu et al., 2020; Wan et al., 2021). The epiphytic yeasts (3.72 log cfu/g FM) on *S. arundinaceum* were higher than LAB (1.29 log cfu/g FM).

**TABLE 1:** Microbial and chemical characterization of unfermented *Saccharum arundinaceum* (mean ± SEM, *n* = 3).

Item	Fresh *Saccharum arundinaceum*
Dry matter (g/kg)	284.23 ± 3.27
Crude protein (g/kg DM)	104.13 ± 3.31
Neutral detergent fiber (g/kg DM)	672.51 ± 1.86
Acid detergent fiber (g/kg DM)	302.32 ± 1.03
Water soluble carbohydrates (g/kg DM)	54.76 ± 0.71
Lactic acid bacteria (log cfu/g FM)	1.29 ± 0.11
Yeasts (log cfu/g FM)	3.72 ± 0.25

DM, dry matter; cfu, colony-forming units; FM, fresh matter.

### Chemical characteristics of *Saccharum arundinaceum* after ensiling

**FIGURE 1:**
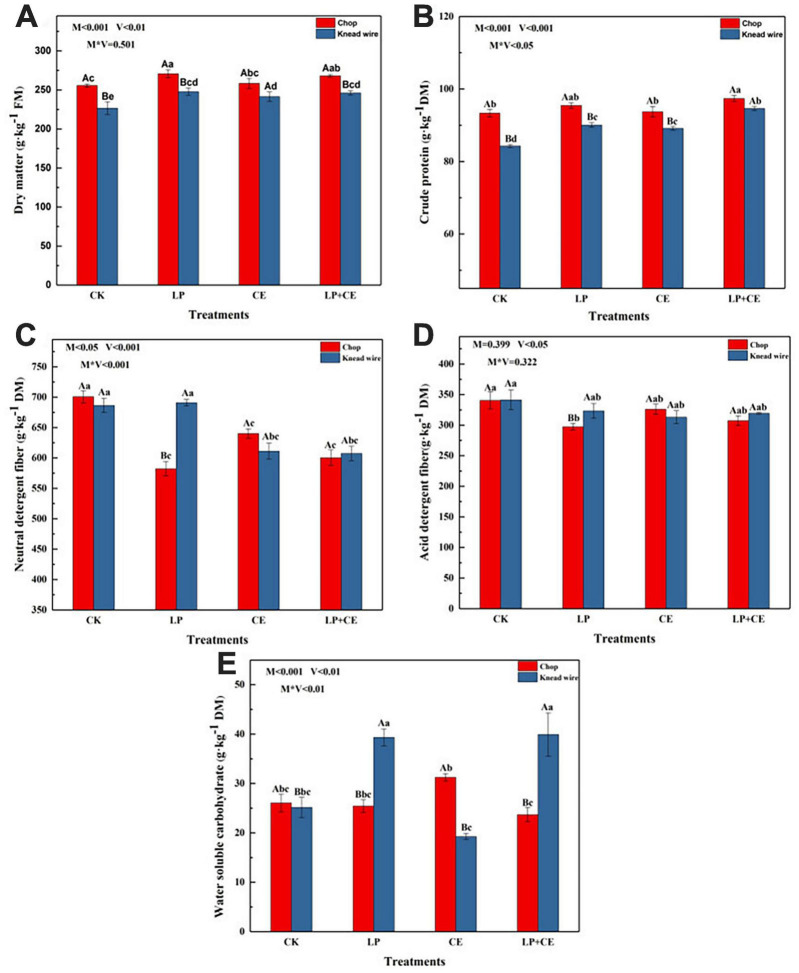
Effects of cutting methods and fermentation additives on **(A)** DM, **(B)** CP, **(C)** NDF, **(D)** ADF, and **(E)** WSC of *Saccharum arundinaceum* after 60 day of ensiling. CK, control; LP, *Lactobacillus plantarum*; CE, cellulase; LP + CE, *L. plantarum* plus cellulase. Significant differences (*P* < 0.05) between the same additive treatments are indicated by capital letters, and significant differences (*P* < 0.05) among different treatments are indicated by lowercase letters. DM, dry matter; FM, fresh matter; M, cutting methods; V, additive; V*M, the interaction between additive and cutting methods.

The effects of cutting methods and additives on the chemical characteristics (DM, CP, NDF, ADF, and WSC contents) of *S. arundinaceum* silage on day 60 are shown in Figure 1. As shown in Figure 1A, the contents of DM in the silages with two additives were well preserved and were significantly higher (*P* < 0.05) than those of the CK treatments (except for the CE-chopped silage). This result was consistent with previous studies showing that adding LP and CE could suppress the growth and/or activity of undesirable microorganisms by rapidly decreasing the pH (Li F. et al., 2019; Wang Y. et al., 2019). The DM content of the chopped treatments was higher than that of the knead-wired silages (*P* < 0.001), which could be attributed to the lower pH of the chopped treatments (Table 2), and more nutrient substances were preserved. Another reason may be that the knead wire could provide high levels of fermentable carbohydrates by its more complex processing method, and epiphytic microorganisms consume much more biomass during ensiling, especially undesirable bacteria (Kõiv et al., 2019). The variation in CP content in *S. arundinaceum* silages (Figure 1B) was similar to that of the DM content, and adding LP + CE also led to higher CP contents than that of the CK-treated silages with chopping method (*P* < 0.05). LP and CE inoculation provided abundant LAB and increased the fermentation material, respectively, which induced the accumulation of organic acids and a quick decrease in pH. The relatively low pH inhibited undesirable microorganism growth and activity and/or proteolytic enzyme activity (Ni et al., 2017; Wang C. et al., 2019); hence, more crude protein in the silages was preserved. The above reason also contributing to the higher CP contents in chopped silages compared to knead wired silages. Moreover, the interaction between additives and cutting methods had a statistically significant effect on the preservation of CP (*P* < 0.05). Therefore, LP + CE-chopped silages had a better effect on preserving the CP.

**TABLE 2:** Fermentative profile of *Saccharum arundinaceum* silage subjected to different cutting methods and additives during fermentation.

Items	Ensiling days (D)	Treatment								Treatment mean	SEM	*P*-value					
		Cutting method (Chop)				Cutting method (Knead wire)						V	M	D	V × M	V × D	M × D
		CK	LP	CE	LP + CE	CK	LP	CE	LP + CE								
pH	3	6.03aC	4.94bF	6.06aC	4.85aF	6.55aA	5.76aD	6.20aB	5.57aE	5.75a	0.05	***	***	***	***	***	***
	7	5.93aB	4.65cE	5.81bB	4.82aD	6.24bA	5.50bC	5.83bB	4.85bD	5.45ab							
	15	5.94aB	5.07aE	5.76bC	4.43cG	6.19bA	5.44bD	5.57cD	4.93bF	5.41ab							
	30	6.03aB	4.92bE	5.65bC	4.52bF	6.28bA	5.12cD	5.57cC	4.95bE	5.38b							
	60	5.29bD	4.87bE	5.44cC	4.51bcF	6.03cA	5.20cD	5.59cB	4.94bE	5.24b							
Lactic acid (g⋅kg^–1^ DM)	3	12.65bcC	27.30bB	10.704e	29.30bA	2.94dE	8.38cD	7.37bD	7.51eD	13.27b	1.57	***	***	***	***	***	***
	7	15.86aC	34.49aA	13.14dD	29.81bB	4.29cdE	16.18bC	12.86aD	27.37bB	19.25a							
	15	14.26abCD	28.40bB	17.04cC	41.49aA	4.64cE	16.59bC	11.91aD	29.21aB	20.44a							
	30	13.86abE	27.34bA	25.45aAB	24.62cB	7.17bF	22.03aC	12.53aE	17.79cD	18.85a							
	60	10.66cD	10.87cD	19.32bB	30.75bA	9.15aD	15.88bC	10.82aD	15.30dC	15.34ab							
Acetic acid (g⋅kg^–1^ DM)	3	4.95cB	5.99bA	3.80cC	4.24eBC	5.03cB	4.38dBC	1.76cD	6.59cA	4.59d	0.04	***	***	***	***	***	***
	7	7.03aB	7.28aB	5.81bC	5.55dC	5.93cC	6.15cC	9.47bA	9.19bA	7.05c							
	15	5.94bC	6.32abC	6.37bC	6.24cC	9.70bB	6.87bC	12.19aA	9.30bB	7.87bc							
	30	5.45bcD	7.42aC	8.03aBC	7.54bC	9.17bB	9.13aB	11.37aA	11.48aA	8.70b							
	60	7.23aE	6.95abE	8.56aD	10.06aC	13.72aA	8.74aD	12.13aB	12.12aB	9.94a							
Lactic acid/Acetic acid	3	2.56bC	4.69aB	2.82abC	6.94aA	0.59bcE	1.92bCD	4.30aB	1.14cDE	3.12a	0.11	***	***	***	***	***	***
	7	2.26bD	4.73aB	2.29bD	5.37bA	0.72abF	2.63aCD	1.37bE	2.99aC	2.80a							
	15	2.42bD	4.49abB	2.68abCD	6.67aA	0.48cF	2.42aD	0.97bE	3.14aC	2.91a							
	30	2.55bB	3.69bA	3.22aA	3.27cA	0.79aD	2.41aB	1.10bCD	1.57bC	2.33ab							
	60	1.48aCD	1.56cCD	2.30bB	3.06cA	0.67abcF	1.83bC	0.90bEF	1.26bcDE	1.63b							
Propionic acid (g⋅kg^–1^ DM)	3	0.25cC	0.12bC	0.23eC	0.20cC	0.11dC	1.46dA	0.12dC	0.52bB	0.38c	0.02	***	***	***	***	***	***
	7	0.39cD	1.25abD	1.23dD	1.65bCD	2.64bBC	3.14cB	4.51aA	3.07aB	2.24b							
	15	1.13bD	1.24abD	2.70cC	1.64bD	5.14aB	6.70bA	4.47aB	3.02aC	3.26ab							
	30	1.31bE	2.43aCD	3.31bB	1.72bDE	1.34cE	9.93aA	3.75bB	3.19aBC	3.37a							
	60	4.02aA	2.71aB	4.08aA	3.71aA	1.45cC	2.82cB	1.69cC	3.57aA	3.01ab							
Butyric acid (g⋅kg^–1^ DM)	3	0.16dE	0.05eF	0.37eB	0.31dC	0.70eA	0.16dE	0.22dD	0.09cE	0.26e	1.31	***	***	***	***	***	***
	7	0.82cC	1.21dA	086dBC	0.46dD	1.00dB	0.54cD	0.71cC	0.18cE	0.72d							
	15	2.029bA	2.13cA	1.37cCD	1.22cDE	1.70cB	1.29bCDE	1.50bBC	1.07bE	1.54c							
	30	3.38aA	2.95bB	2.34bD	2.75bC	1.89bE	1.20bG	1.60abF	1.43aF	2.19b							
	60	3.41aB	3.93aA	3.32aB	3.56aB	2.98aC	1.75aD	1.71aD	1.42aE	2.76a							
NH_3_-N (g⋅kg^–1^ TN)	3	12.34dC	14.51cBC	14.77eBC	12.91dC	20.40dA	15.11dBC	17.16dAB	14.82dBC	15.25e	9.48	***	***	***	***	**	***
	7	68.10cA	31.80cCDE	36.03dCD	29.16dDE	40.16cC	23.49dEF	56.35cB	19.74dF	38.10d							
	15	125.24bA	121.30bAB	128.20cA	117.58cAB	117.18bAB	99.93cB	72.5bc4C	72.53cC	106.81c							
	30	123.10bB	140.43bAB	154.17bA	146.09bA	151.27aA	123.11bB	87.36bC	99.99bC	128.19b							
	60	172.38aA	161.87aA	172.66aA	161.08aA	162.01aA	140.92aB	139.44aB	134.17aB	155.57a							

CK, control; LP, *Lactobacillus plantarum*; CE, cellulase; LP + CE, *L. plantarum* plus cellulase. Significant differences (*P* < 0.05) between the same additive treatments on the different ensiling d are indicated by lowercase letters. Significant differences (*P* < 0.05) between the different treatments on the same ensiling d are indicated by capital letters; SEM, standard error of means; DM, dry matter; TN, total nitrogen; V, additive; M, cutting method; D = ensiling day; V × M, the interaction between additive and cutting method; V × D, the interaction between additive and ensiling day; M × D, the interaction between cutting method and ensiling day; ***P < 0.001, **P < 0.01.

As shown in Figures 1C,D, M (cutting methods) × V (additives) significantly influenced the content of NDF (*P* < 0.001) and V (additives) significantly influenced the ADF content (*P* < 0.05). The NDF contents in the CE- and LP + CE-treated *S. arundinaceum* silages were lower than those of the CK (*P* < 0.05), while there was no significant difference (*P* > 0.05) between CE and LP + CE. Cellulase can catalyze the hydrolysis of cellulose; therefore, the CE- and LP + CE-treated silages showed a large reduction in NDF content (Xu et al., 2020; Du et al., 2021). Moreover, the NDF contents of LP-chopped silages were also significantly decreased compared with those of CK-chopped silages, but LP-kneaded silages showed no difference from those of the CK. The above result was probably due to the pH lower than 5.0 of LP-chopped silages in most of the fermentation time (Table 2), while LP-kneaded silages had a higher pH than LP-chopped treatments. The relatively low pH led to acid hydrolysis, which reduced the lignocellulosic structure (Yang et al., 2019). For ADF contents, all the treatments had no significant difference from CK, except for the LP-chopped silages. Lignin and hemicellulose constitute lignin-carbohydrate complexes that act as a protective layer to prevent cellulose degradation (Yu et al., 2005), which may result in high levels of NDF degradation and low levels of ADF degradation in *S. arundinaceum* silages. Another reason for this observation may be that ADL (included in the ADF) is difficult to degrade, and *S. arundinaceum* has high levels of lignin; moreover, lignin is closely associated with hemicellulose by ester and hydrogen bonds (Li et al., 2018).

Water-soluble carbohydrates are the major fermentation substrates for LAB during ensiling and can be converted into organic acids and rapidly decrease the pH of silages by LAB (Ahmadi et al., 2019; Xu et al., 2020). The influence of different cutting methods and additives on the WSC contents of *S. arundinaceum* silage is shown in Figure 1E. The WSC content of CE-chopped silages following 60 day of ensiling was higher than that of other silages, but only significantly higher than LP + CE-chopped silages. These results indicate that the addition of CE improved lignocellulosic degradation and preserved more WSCs (Xu et al., 2020). The lower WSC contents of LP + CE silages than CE silages were probably because the addition of LP enhanced the abundance of LAB to consume more WSCs, corresponding to the result of bacterial community abundance, in which the relative abundance of *Lactobacillus* in CE and LP + CE silages was 0.27 and 80.38%, respectively (Figure 3). In general, the interaction of additives × cutting methods existed for the WSC contents (*P* < 0.01). Moreover, for LP and LP + CE, the knead-wired silages had higher WSC contents, had higher pH values and poorer fermentation quality than chopped silages (Table 2), which needs further study.

**FIGURE 2:**
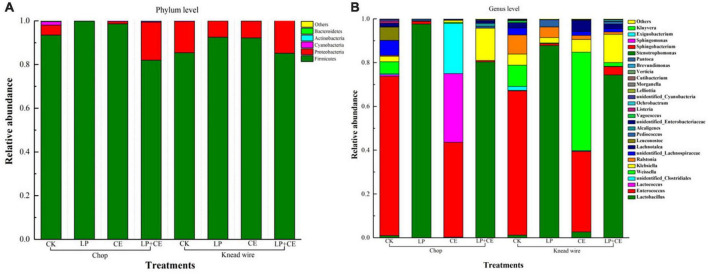
Taxonomic classification and relative abundances of the bacterial community of 60-day *Saccharum arundinaceum* silage. Bacteria were identified to the **(A)** phylum and **(B)** genus levels. CK, control; LP, *Lactobacillus plantarum*; CE, cellulase; LP + CE, *L. plantarum* plus cellulase.

**FIGURE 3:**
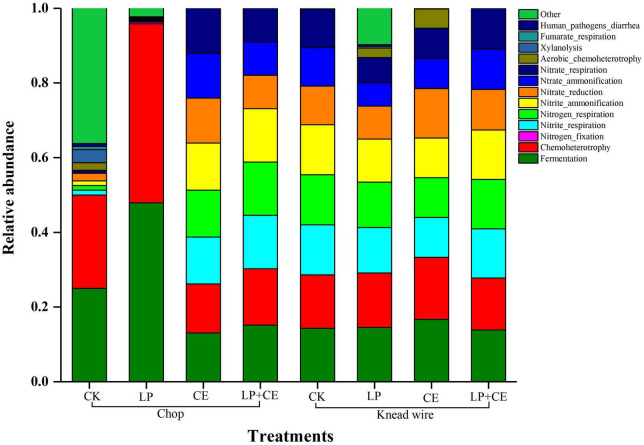
Functional prediction of the bacterial community of 60-day *Saccharum arundinaceum* silage. CK, control; CE, cellulase; LP, *Lactobacillus plantarum*; LP + CE, combination of *L. plantarum* and cellulase. CK, control; LP, *L. plantarum*; CE, cellulase; LP + CE, *L. plantarum* plus cellulase.

### Fermentation profile and microbial population of *Saccharum arundinaceum* silages

The effects of cutting methods and additives on the silage fermentation profile of *S. arundinaceum* are listed in Table 2. In our study, the interaction of V × M existed for pH (*P* < 0.001), concentrations of LA (*P* < 0.001), AA (*P* < 0.001), PA (*P* < 0.001), BA (*P* < 0.001), and NH_3_-N (*P* < 0.001), and LA/AA (*P* < 0.001).

Silage pH is an important indicator for ensiling (Larsen et al., 2019; He et al., 2020). During fermentation, the pH value of LP and LP + CE-chopped silages decreased to 4.94 and 4.85 in the first 3 day and continuously decreased to 4.87 and 4.51 on day 60, respectively, which were significantly lower (*P* < 0.05) than those of the other treatments on each ensiling day. Therefore, LP addition accelerated the pH decline. As expected, adding LP (LP and LP + CE treatments) to the chopped and knead-wired silages led to a lower pH than that of the CK groups (*P* < 0.05), as LP addition could improve fermentation by producing abundant lactic acid (Li F. et al., 2019; Guo et al., 2021). Moreover, compared with LP treatments, the pH value of LP + CE silages significantly decreased (except for the chopped silages on day 3), which might be because the addition of CE improved the efficiency of cellulose degradation, thereby increasing the substrate available for LAB fermentation (Xu et al., 2020). For the same additive treatment, kneaded wired silages had higher pH values than chopped silages, which was probably due to the lower concentration of lactic acid and the higher abundance of *Weissella* and lower abundance of *Lactobacillus* (Mu et al., 2020; Ren et al., 2020; Du et al., 2021).

In Table 2, the dynamics of the organic acid concentration of *S. arundinaceum* silages are shown. Inoculation with LP significantly (*P* < 0.05) increased the silage contents of LA subjected to both cutting methods (except for the chopped silages on day 60). *L. plantarum* is known to potentially improve fermentation quality and reduce silage pH (Ni et al., 2017), which could contribute to the lower pH values of both the LP and LP + CE treatments. In contrast to the silage pH value, knead-wired silages have a lower concentration of LA than chopped silages, especially at the initial fermentation. The AA concentration was higher in *S. arundinaceum* silages with knead wire (especially in the CE groups) vs. chopped silages (*P* < 0.001). The major reason for the higher AA concentration in knead wire-treated *S. arundinaceum* silages could be attributed to the enhancement of heterolactic acid fermentation (Li F. et al., 2019). The reason for this phenomenon may be that heterofermentative microorganisms are dominant in tropical forages of spontaneous fermentation form, as previously reported (Shao et al., 2003). The addition of CE to knead wire-treated silages may lead to a higher relative abundance of *Weissella*, which are obligatory heterofermentative LAB (Figure 2; Graf et al., 2016; Ren et al., 2020). As expected, inoculation with LP increased the ratios of LA/AA of the silages compared to those of the CK- and CE-treated silages (*P* < 0.001), which could be illustrated by the addition of homofermentative LAB (*L. plantarum*), increasing the homolactic fermentation and LA production of the silage (Lin et al., 2020).

For the concentration of PA and BA, adding LP resulted in lower concentrations of these two organic acids (except for PA in knead-wired silages) (*P* < 0.05). Generally, excess moisture can impair silage fermentation and secondary fermentation by *Clostridia* and *Enterobacteria*, which can convert LA into PA (Kung, 2008). Adding LP (LP and LP + CE) significantly reduced the NH_3_-N content compared with the CK and CE-treated silages (*P* < 0.05). The above phenomenon showed that LP silage inhibited the proteolytic action of plant enzymes and the growth and/or activity of *Clostridia* and *Enterobacteria* (Figure 3; Guan et al., 2018; Cai et al., 2020; Dong et al., 2020), which can be found in Figure 3. In contrast, a lower content of NH_3_-N was detected in knead-wired silages than in chopped silages (*P* < 0.001); further studies are required to explore this phenomenon.

The dynamics of the LAB and yeast populations of *S. arundinaceum* silages are listed in Table 3. The number of LAB increased at the initial stage and then decreased from day 7 (Treatment mean), as many LAB strains are relatively intolerant to lower pH values (Ohmomo et al., 2002). Although no significant difference (*P* > 0.05) was observed in LAB populations between chopped and knead-wired silages, additives had a strikingly significant (*P* < 0.001) impact on the LAB population in *S. arundinaceum* silage. Before 15 days of fermentation, adding LP resulted in lower numbers of yeast because of the decrease in pH. Yeast was not detected in any of the samples after 30 days, which was due to the anaerobic environment during the late period of silage fermentation (Zhang et al., 2021), indicating better fermentation performance.

**TABLE 3:** Microbial population of *Saccharum arundinaceum* subjected to different cutting methods and additives during fermentation.

Items	Ensiling days (D)	Treatment								Treatment mean	SEM	*P*-value					
		Cutting method (Chop)				Cutting method (Knead wire)						V	M	D	V × M	V × D	M × D
		CK	LP	CE	LP + CE	CK	LP	CE	LP + CE								
LAB (cfu⋅g^–1^ FM)	3	5.43cB	6.78bA	5.66dB	6.38cA	5.70cB	6.71bcA	6.64bA	6.62bA	6.24c	0.05	***	0.26	***	0.18	***	***
	7	6.90aD	7.33aBC	7.49aAB	7.43aABC	7.18aCD	7.50aAB	7.67aA	7.49aAB	7.37a							
	15	6.53abBC	6.68bcAB	6.80bAB	6.85bA	6.40bC	6.89bA	6.73bAB	6.39bcC	6.66b							
	30	6.08bBC	5.87dCD	6.23cB	6.56bcA	6.17bcBC	6.56cA	5.66cD	6.55bA	6.21c							
	60	6.12bAB	6.39cAB	6.18cAB	6.62bcA	6.02bcAB	6.12dAB	5.92cB	6.30cAB	6.21c							
Yeasts (cfu⋅g^–1^ FM)	3	6.76aB	5.46aD	7.60aA	6.39bC	7.28aA	2.29bE	6.39bC	5.14bD	5.91a	0.02	***	***	***	***	***	***
	7	6.32bC	3.64bE	6.39bC	6.85aB	4.42bD	2.24bF	7.25aA	7.05aAB	5.52a							
	15	3.78cA	1.75cE	3.26cB	2.70cC	2.54cCD	2.52aCD	1.39cF	2.30cD	2.53b							
	30	ND	ND	ND	ND	ND	ND	ND	ND	–							
	60	ND	ND	ND	ND	ND	ND	ND	ND	–							

CK, control; LP, *Lactobacillus plantarum*; CE, cellulase; LP + CE, *L. plantarum* plus cellulase. Significant differences (*P* < 0.05) between the same additive treatments on the different ensiling d are indicated by lowercase letters. Significant differences (*P* < 0.05) between the different treatments on the same ensiling d are indicated by capital letters; SEM, standard error of means; cfu, colony-forming units; FM, fresh matter; ND, not detected; V, additive; M, cutting method; D = ensiling day; V × M, the interaction between additive and cutting method; V × D, the interaction between additive and ensiling day; M × D, the interaction between cutting method and ensiling day; “–” represents Not analyzed; ***P < 0.001.

### Bacterial community diversity of *Saccharum arundinaceum* silage

The observed species, Good’s coverage, alpha diversity including ACE, Chao1 estimation, and Shannon index of bacteria are summarized in Table 4. Alpha diversity reflects the bacterial abundance (ACE and Chao1) and species diversity (Shannon index) in a sample (Du et al., 2021). After ensiling, the LP- and CE-inoculated chopped silage produced lower observed species, ACE, Chao1 estimation, and Shannon index than the CK silages, possibly due to the lower pH in the additive treatments, which could inhibit the growth of some microorganisms (Méndez-García et al., 2015). In contrast, CE-treated knead-wired silages had greater species richness, abundance (ACE and Chao1), and diversity (Shannon index) than CE-chopped silages. Knead-wired silages tended to have an increased pH, likely conducive to the survival of many microbes, including heterofermentative LAB (Hu et al., 2009; Yang et al., 2019). Good’s coverage of all samples was above 0.99, the depth of sequencing results which can represent the real situation of the silage samples well and makes it feasible to analyze the microbial community (Yang et al., 2019).

**TABLE 4:** Bacterial species richness and diversity of *Saccharum arundinaceum* silage subjected to different cutting methods and fermentation additives.

Treatment		Observed species	ACE	Chao1	Shannon	Good’s coverage
Chop	CK	26ab	34.88a	35.33a	2.30a	0.994c
	LP	16cd	17.15cd	16.50bc	1.22bc	0.998a
	CE	11d	13.45d	11.51c	0.78c	0.998a
	LP + CE	26a	32.96ab	32.94a	1.99ab	0.995bc
Knead wire	CK	20abcd	24.59abcd	21.98abc	2.19a	0.997ab
	LP	17bcd	20.57bcd	24.27abc	0.98c	0.997ab
	CE	22abc	27.79abc	24.87abc	2.06ab	0.996abc
	LP + CE	28a	33.30ab	31.29abc	2.44a	0.996bc
SEM		1.42	2.00	2.09	0.15	0.002
*P*-value		**	*	*	**	*

CK, control; *LP, Lactobacillus plantarum*; CE, cellulase; LP + CE, *L. plantarum* plus cellulase; SEM, standard error of means. Significant differences (P < 0.05) among the different treatments are indicated by lowercase letters; **P < 0.01, *P < 0.05.

The bacterial community structure in the *S. arundinaceum* silage on day 60 was explored by high-throughput Illumina MiSeq sequencing. As shown in Figure 2, the phylum and genus levels of bacterial community compositions are visualized as stacked columns. Figure 2A shows that the silages were dominated by two main phyla (Firmicutes and Proteobacteria) at the end of ensiling, which can be commonly found in previous studies (Mu et al., 2020; Ren et al., 2020). Firmicutes, which is crucial to producing organic acids such as LA and AA at the later stage of fermentation (Zhao et al., 2017), became the most dominant bacterial phylum in all the samples, with a relatively high abundance of 82%∼99.8%, especially in the LP- and CE-chopped silages (99.8 and 98.62%, respectively). For knead-wired silages, the higher relative abundance of Proteobacteria, which can digest organic matter, was probably due to a variety of factors, including climate and silage material composition (Guan et al., 2018; Mu et al., 2020; Yuan et al., 2020). In our study, the reason may be the cutting methods.

In Figure 2B, the most predominant genera in the CK treatments of the chopped and knead-wired silages were *Enterococcus* (72.74 and 65.93%, 0.31%, respectively), *Weissella* (5.46 and 9.74%, respectively) and *Klebsiella* (2.46 and 5.06%, respectively), and in the CE treatments were *Enterococcus* (43.3 and 36.77%, respectively), *Lactococcus* (31.43 and 0.09%, respectively), unidentified_ *Clostridiales* (22.93 and 0.31%, respectively), Weissella (0.51 and 45.01%, respectively). The dominant genera in the LP and LP + CE treatments of the two cutting methods were *Lactobacillus* (74.37∼97.65%) and *Klebsiella* (0∼12.85%), while chop-treated silages had a relatively higher abundance of *Lactobacillus* and a relatively lower abundance of *Klebsiella*. The LAB genera *Lactobacillus*, *Weissella*, and *Lactococcus* are functional bacteria that can be used to improve silage quality (Yang et al., 2016). Moreover, *Lactobacillus* played a vital role in silage fermentation, as they could decrease the pH of silage rapidly to preserve the nutrient substance and could remain active and viable at low pH values (Dunière et al., 2013; Zhang et al., 2021). Therefore, adding LP significantly enhanced *Lactobacillus* abundance, which resulted in the lower silage pH of the LP and LP + CE treatments of the chopped and knead-wired silages compared with that of the CK and CE groups, which is in line with the findings of previous studies (Ren et al., 2020; Zhang et al., 2021). Relatively speaking, *Enterobacter* are considered undesirable bacteria in silage, as they may compete with LAB to utilize fermentation substrates and produce NH_3_-N. As a result, the high relative abundance of *Enterobacter* in the CK and CE treatments led to a slower decline in pH and an increase in protein degradation (Wang Y. et al., 2019), which is shown in Table 2 and Figure 1. Therefore, for LP and LP + CE, especially in chopped silages, undesirable growth of microorganisms of *Enterobacter* was inhibited, and the dominant *Lactobacillus* growth was increased, which resulted in a low pH value in the silage (Dunière et al., 2013). A higher *Weissella* abundance was found in the CK- and CE-knead wire-treated silages compared with that of other knead wired silages, which belong to the heterofermentative LAB and consume WSCs to produce both LA and AA, leading to an increasing concentration of AA (Table 2; Graf et al., 2016). *Lactococcus* were identified at the early stage of silage fermentation and exhibited a declining trend as fermentation proceeded, which decreased gradually due to the rapid drop in pH value and severe acidic conditions (Liu B. Y. et al., 2019). Bacteria from the genus *Klebsiella* can convert amino acids into ammonia and biogenic amines (Zhang et al., 2021), and the higher abundance of *Klebsiella* probably resulted in the lower CP content of knead wire-treated silages (Figure 1).

### Function prediction and correlation analysis of the microbial community based on some silage parameters

Functional prediction of the bacterial community of *S. arundinaceum* silage fermentation on day 60 was visualized as a stacked column (Figure 3). Fermentation was the main function of the microorganism community, followed by chemoheterotrophy, nitrite respiration, nitrogen respiration and nitrite ammonification. The bacterial communities of chopped silages had a higher proportion of fermentation and chemoheterotrophy functions compared with knead-wired silages, indicating that carbon fixation was inhibited and energy was obtained through organic oxidation in these silages (Zhang et al., 2018). Therefore, chopped treatment could enhance ensiling fermentation, resulting in lower pH values (Table 2), which were favorable for silage preservation. The LP-treated chopped silages promoted the fermentation of the bacterial community, whereas LP + CE-treated chopped silages had no significant effect vs. the CK-chopped silages. The deceased rate and extent of fermentation of the treatment with LP + CE need further study. Moreover, the high levels of nitrite respiration, nitrogen respiration and nitrite ammonification of the bacterial community in knead-wired silages could lead to lower CP contents (Figure 1; Li et al., 2021).

**FIGURE 4:**
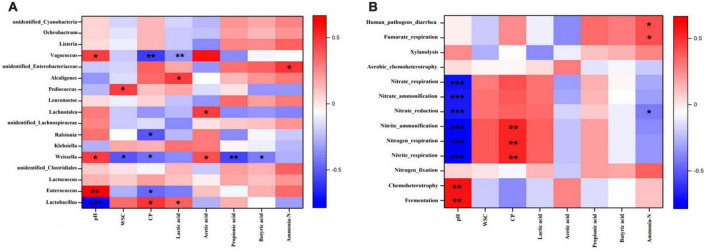
Heatmap of Spearman correlations between bacterial community **(A)** predicted functions **(B)** and silage characteristics of 60-day *Saccharum arundinaceum* silage. Heatmap colors denote the Spearman correlation coefficient “*r*” (–1 to 1). *r* > 0 represents a positive correlation, and *r* < 0 represents a negative correlation. The asterisks *, **, and *** represent statistical significance levels of *P* < 0.05, < 0.01 and < 0.001, respectively.

As shown in Figure 4, the interactions between some silage parameters and bacterial community/function prediction of the bacterial community in the *S. arundinaceum* silages were presented by establishing their Spearman correlations and then visualized in heatmaps. Figure 4A shows the correlations between the metabolites and microbial diversity. In general, silage fermentation metabolites can be improved by the diversity of microorganisms, and the produced metabolites can affect the bacterial community and thus silage quality (Dong et al., 2020; Li et al., 2021). Therefore, many previous studies have investigated the correlations between microorganisms and silage fermentation parameters (Xu et al., 2018; Ren et al., 2020; Li et al., 2021). For example, Li et al. (2021) found that the bacteria in the genus *Pediococcus* were negatively correlated with pH value and positively correlated with the LA concentration in silage, which was consistent with our findings. In our study, the dominant genus *Lactobacillus* was positively correlated with the contents of CP (*r* = 0.494, *P* < 0.05) and LA (*r* = 0.494, *P* < 0.05) and negatively correlated with pH (*r* = −0.765, *P* < 0.001) in the *S. arundinaceum* silage. However, the relative abundance of *Enterococcus* was positively correlated with pH (*r* = 0.656, *P* < 0.001) and negatively correlated with CP content (*r* = −0.458, *P* < 0.05). These results indicated that inoculation with *L. plantarum* enhanced the concentration of LA, which decreased the pH and inhibited undesirable microorganisms during ensiling to preserve the silage nutrient substance (e.g., CP and DM) (Dong et al., 2020; Ren et al., 2020). *Weissella* can produce both LA and AA (Yan et al., 2019); therefore, pH (*r* = 0.453, *P* < 0.05) and AA concentration (*r* = 0.425, *P* < 0.05) were significantly positively correlated with *Weissella* abundance, probably slowing the pH decline and thus resulting in unfavorable silage fermentation. Therefore, the contents of CP (*r* = –0.431, *P* < 0.05), WSC (*r* = –0.503, *P* < 0.05), PA (*r* = –0.571, *P* < 0.05) and BA (*r* = –0.407, *P* < 0.05) were significantly negatively correlated with *Weissella* abundance. The relationships between the ensiling characteristics and functional prediction in the bacterial community are shown in Figure 4B. Silage pH was significantly positively correlated with fermentation and chemoheterotrophy (*P* < 0.01). The CP contents were highly positively correlated with nitrite respiration (*r* = 0.537, *P* < 0.01), nitrogen respiration (*r* = 0.537, *P* < 0.01) and nitrite ammonification (*r* = 0.532, *P* < 0.01) of the bacterial community, which could result in the lower CP content. This phenomenon can be found in Figure 1.

## Conclusion

The results showed that adding LP and CE efficiently increased the content of DM, CP, and LA and decreased the content of NDF and pH of *S. arundinaceum* silage, especially chop-treated silage. Moreover, the bacterial community structure underwent great changes with different cutting methods and additives, with increased abundance of *Lactobacillus* in the LP and CE-chopped treatment. In summary, the chopped *S. arundinaceum* silages treated with the LP + CE inoculant achieved better fermentation quality and preservation of nutrient substances. Therefore, *S. arundinaceum* can serve as a new silage resource for feed utilization by the ensiling method of LP + CE-chopped.

## Data availability statement

The raw data supporting the conclusions of this article will be made available by the authors, without undue reservation.

## Author contributions

YZ, PL, and CC designed the study, wrote the manuscript, and were involved in the revision of the manuscript. YZ, PL, ML, JX, and HS performed the experiments. QC, YX, CW, and PL conducted the statistical analysis. All authors reviewed and approved the final manuscript.

## Footnotes

1
https://www.arb-silva.de/

2
https://www.originlab.com/
